# Can the implementation of household waste classification mitigate greenhouse gas emissions in Beijing? A comprehensive analysis of recent trends and future scenarios

**DOI:** 10.1016/j.heliyon.2023.e23132

**Published:** 2023-12-01

**Authors:** Zhixin Wen, Huimin Li, Yufei Wang, Xiaofan Zhao, Xianghui Deng

**Affiliations:** a Beijing Climate Change Response Research and Education Center, Beijing University of Civil Engineering and Architecture, Beijing, 100044, China; b Department for Consulting and Research, Management World Journal, Beijing, 100026, China; c Division of Public Policy, The Hong Kong University of Science and Technology, Hong Kong, China; d China Industrial Energy Conservation and Cleaner Production Association, Beijing, 100034, China

**Keywords:** Household waste classification, GHG emissions, Reduction potential, Carbon neutral

## Abstract

Household waste contributes significantly to global greenhouse gas (GHG) emissions, and waste classification is crucial for reducing emissions. This study focuses on Beijing and utilizes life cycle assessment (LCA) and material flow analysis (MFA) to calculate GHG emissions in waste management systems and quantify emission reduction potential of different measures. The results show that net emissions from the classification system in 2021 are 116.77 kg CO_2_-eq/t waste, reducing 61.82 % compared to the traditional mixed collection and transportation system. Waste volume, classification efficiency, and treatment strategies are the primary factors affecting emissions in classification systems. Recycling is identified as effective treatment methods. Three scenarios are designed to explore emission pathway of the system toward 2060. In the business-*as*-usual (BAU) Scenario, emissions will continue to grow to 108.57 × 10^4^ t CO_2_-eq/yr in 2060. In the Classification Efficiency Scenario and the Comprehensive Scenario, emissions in 2060 will be cut to −177.26 × 10^4^ t CO_2_-eq/yr and −702.00 × 10^4^ t CO_2_-eq/yr, respectively. These results underscore the critical role of waste classification and recycling in mitigating the negative impacts of increasing waste volume. By 2060, combining waste classification with recycling can offset emissions by 803.51 × 10^4^ t CO_2_-eq/yr, contributing 99 % to emission reduction potential. Improving classification efficiency and recycling ratio are key measures for achieving this reduction goal. Meanwhile, treatment methods and technologies should prioritize classification and recycling. Aiming at carbon neutrality, the study proposes several recommendations to improve classification systems, including enhancing classification efficiency, optimizing treatment facilities and strategies, and establishing recycling and utilization systems, etc.

## Highlights

• Exploring the emission trajectories of household waste system in different scenarios toward 2060.
• Quantifying the emission reduction potential of transitioning from a mixed waste system to a classification system.
• Putting forward the emission reduction roadmap from material recycling, disposal methods and technological improvements.

## Graphical abstract

**Figure:**
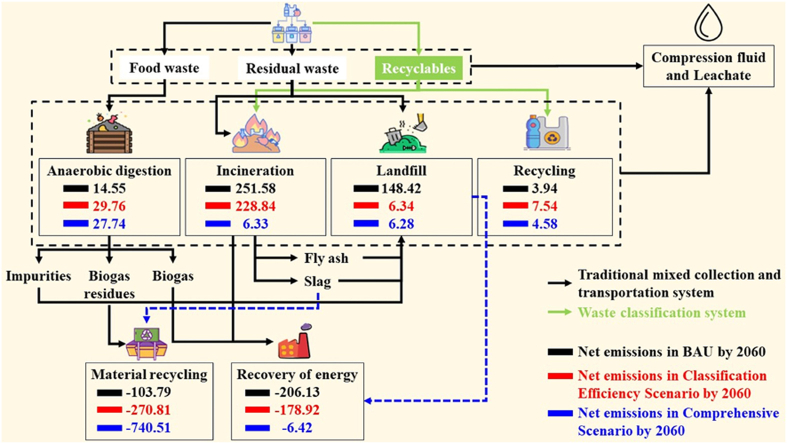

## 1 Introduction

GHG from waste has been recognized as one of the major sources of GHG emissions [1,2], accounting for 5 % of the total at the global level [2,3]. In China, emissions reached about 203.54 million tons in 2019, accounting for 1.68 % of total emissions [4], and they can continue to increase due to growing household waste induced by the urbanization, economic development, and increasing consumption [2,3]. Reducing emissions from household waste is a key measure to meeting China's targets for Intended Nationally Determined Contributions (INDC), i.e., achieving a carbon emission peak before 2030 and carbon neutrality before 2060.

Waste classification is widely regarded as a core measure for reducing GHG emissions [2,5]. In theory, the GHG emissions reduction benefits of household waste derive from waste reduction, waste recycling, and household waste treatment strategies [[6], [7], [8], [9], [10]]. On the one hand, waste classification can reduce household waste directly by guiding consumption behavior. On the other hand, waste classification is conducive to resource recycling because sorted waste is always reused as materials or energy [11,12]. Waste classification measures usually involve transforming the waste management system, including collection, transportation, and treatment [13]. It is challenging to quantify the emission reduction benefits of the household waste classification measures as the system is complex [14].

The existing literature has paid attention to the emission reduction potential of different treatment methods, including landfill, anaerobic digestion, and incineration, among others [2,15]. Scholars have further discussed the energy utilization potential and the feasibility of household waste as ‘alternative energy’, such as waste incineration power generation, landfill gas recovery and utilization, and anaerobic digestion [[16], [17], [18], [19]]. For specific materials and organic waste, scholars have also explored the emission reduction potential of material separation, recycling, and reuse [[20], [21], [22], [23], [24]]. Existing literature has laid a good foundation for quantifying the emission reduction benefits of household waste classification measures.

Many scholars have analyzed the impact of waste classification and its treatment on GHG emissions. They have analyzed net emissions under different circumstances. Regarding the waste types, studies mainly have focused on the environmental benefits of separating and changing the treatment of food waste [25]. For example, when food waste was anaerobically digested after classification and other waste were incinerated, the net emissions were 0.0016 t CE/t waste (carbon equivalent, CE), 67.6 % lower than when all waste was incinerated [5]. Additionally, a study also showed that when the classification and collection ratio of paper, plastic, metal, and glass in Nan Province and Luang Prabang (LPB), two cities in the ASEAN region, reached 50%–60 %, GHG emissions could be reduced by 13∼27 × 10^3^ t CO_2_-eq/yr [26]. Furthermore, the emission reduction potential is related to the waste composition and treatment methods. For example, the emission reduction potential in Nan Province was mainly from recycling, while in LPB, it was mainly from composting.

In practical applications, anaerobic digestion, waste incineration, and recycling of recyclables are commonly used after waste classification. In Qingdao, China, the emissions of waste classification and corresponding treatment were −269.3 kg CO_2_-eq/t waste [27]. In Xiao'er Township, Sichuan Province, the GHG emissions were reduced by 55 % after waste classification, reaching 284.09 kg CO_2_-eq/t waste, the net emissions were −116.25 kg CO_2_-eq/t waste after improving the classification efficiency and recovery rate [28]. A study also showed that if waste classification is implemented nationwide, 125 million tons of GHG emissions could be reduced by 2060 [10].

Waste classification has a significant effect on reducing GHG emissions, but the specific effect varies on factors such as region, treatment methods, and classification efficiency. Waste classification has the potential to reduce waste generated at the source, which can help mitigate greenhouse gas emissions associated with waste processing. However, it also has the potential to alter the composition of waste, affecting its physical and chemical properties such as the calorific value, and potentially impacting greenhouse gas emissions during subsequent processing and treatment processes. In addition, due to the change in material flow from waste classification, indirect emissions such as transportation also need to be considered [10]. Overall, waste classification is the key to achieving net-zero emissions in waste management, but its reduction ability still needs further research.

Since 2000, China has started to explore waste classification and announced the first batch of eight pilot cities, including Beijing, Shanghai, Guangzhou, Shenzhen, and other four cities, the pilots have provided important experience for future waste classification efforts. Due to the imperfect policies, regulations, and lack of effective institutional guarantees and implementation, waste classification was hindered. In 2017, the National Development and Reform Commission (NDRC) and the Ministry of Housing and Urban-Rural Development (MHURD) jointly issued *the Implementation Plan for the household waste Classification System*, which required 46 pilot cities to implement household waste classification. This initiative provided policy guarantees and encouraged broad participation across society, resulting in waste reduction, resource recovery, etc. By 2021, over 200 cities in China have implemented waste classification and seen some success. Beijing, as a pilot city, issued *the Opinions of the General Office of Beijing Municipal People's Government on Accelerating the Work of household waste Classification* in 2017. In 2020, *the Regulations of Beijing household waste Management* (hereinafter referred to as *“Regulation”*) were announced, making waste classification compulsory. Beijing's household waste removal was 7.842 million tons in 2021, down from 10.112 million tons in 2019. Nevertheless, the exact impact of waste classification on GHG emissions reduction remains uncertain. Hence, it is necessary to conduct a more comprehensive discussion on Beijing's waste classification system from the perspective of GHG quantification and emission reduction. The household waste management system will help to achieve carbon neutrality.

In conclusion, the emission reduction potential of waste classification systems is dynamic and complex, dependent on factors such as region, waste volume, components, classification efficiency, and treatment strategy. Therefore, compared to previous studies, this study further considers the impacts of components, classification efficiency, and treatment strategies on GHG emissions resulting from waste classification. The life cycle assessment (LCA) approach is employed to evaluate cradle-to-grave (from community collection to treatment) GHG emissions from household waste, expanding the scope of the study. This study provides a quantitative analytical result that can offer decision-making support for the transition of household waste systems towards carbon neutrality.

This paper is organized as follows: Section 2 expounds on household waste composition and waste flow in Beijing. Section 3 presents the methodology of process-based LCA and IPCC guidelines, as well as the framework for scenario setting. Section 4 describes the results of the GHG emissions between the waste classification system and the traditional mixed collection and transportation system in Beijing, China. Section 5 is policy implications. Finally, Section 6 draws the research conclusions.

## 2 Household waste classification in Beijing

In this study, Beijing, with 16 municipal districts is selected as a case. The household waste in this study is generated from the residential, office, and public areas, but excludes the construction waste and the medical waste. The focus is mainly on household waste generated by units and individuals in their daily lives. *The Regulation* has come into force in May 2020. According to *the Regulation*, household waste has been classified into four categories, namely food waste, recyclables, hazardous waste, and residual waste. Each category should be collected, transported, and treated separately.

**Fig. 1:**
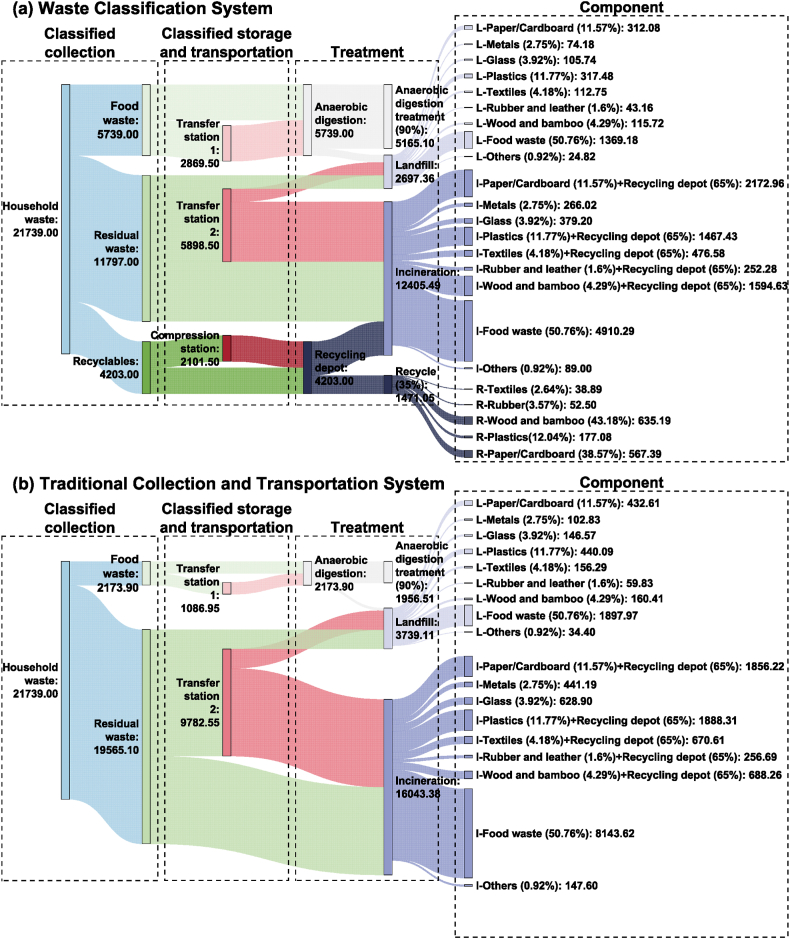
Source and sink of household waste in the waste systems (t/d).

The household waste flow in the waste system is listed as follows (shown in Fig. 1). In the waste classification system (shown in Fig. 1(a)), the household waste is collected from communities and other units first, and then transported, and sent to the treatment facilities. The separated food waste is sent to the anaerobic digestion plant for treatment. Part of the impurities (10 %) [29] are separated in the pretreatment process and then sent to the landfill plant for burial, while the remaining part (90 %) is treated by anaerobic digestion. The residual waste is sent directly for incineration, and after that, the fly ash and slag (10 % each) [5] are sent to the landfill plant for burial (not included in Fig. 1). At present, the recycling rate of recyclables in Beijing is about 35 % [30], which means that 65 % sorted recyclables need to be sent to the incineration plants for treatment. To calculate simply, the waste is divided according to its component ratio.

The traditional mixed collection and transportation system (shown in Fig. 1(b)) is established for comparison. Assume that in this process, only 10 % of food waste is separated and no recyclables are recovered. Other technical conditions, such as the waste components, the efficiency of power generation, biogas utilization, etc., are the same as the waste classification system.

**Fig. 2:**
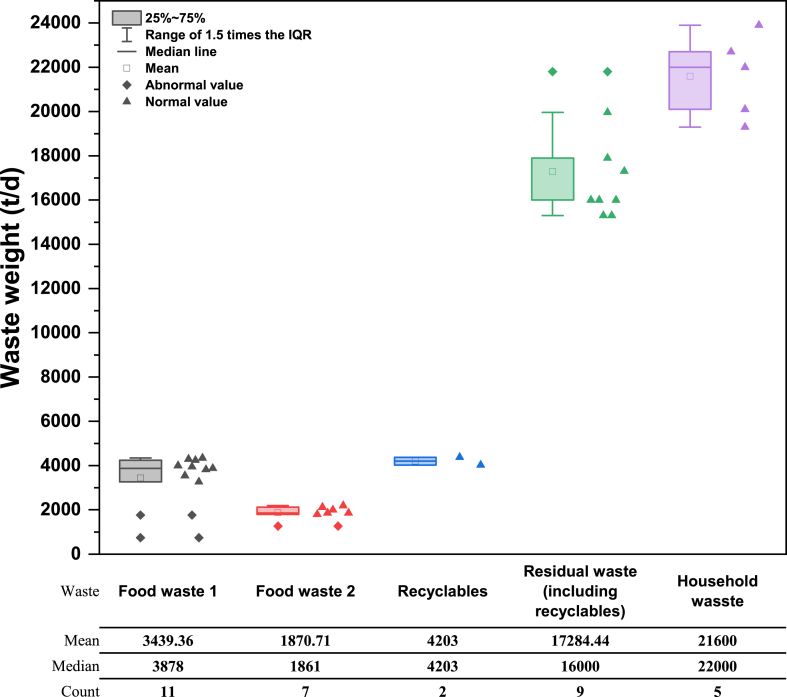
Household waste volume in Beijing in 2020–2021. Note: 1. Waste volume is classified according to the Beijing Municipal Commission of Urban Management. “Food waste 1” refers to waste generated by units, families, communities, etc., while “Food waste 2” refers to restaurant kitchen waste, etc.

In addition, hazardous waste has not been considered in this study as it is generally not disposed of by traditional treatment facilities. Considering the quantity of household waste was reduced after the implementation of *the Regulation*, the study uses the data from May 2020 to July 2021 as an example (shown in Fig. 2). Meanwhile, according to the design capacity of the transfer station (see Table 1) and the household waste amount in 2021 (see Fig. 1), it is assumed that the indirect and direct transportation ratio of household waste is 1:1.

2. In this study, the term “food waste” encompasses both “Food waste 1” and “Food waste 2”. “Residual waste” refers to the portion that excludes recyclables. “Household waste” refers to the sum of “food waste”, “recyclables” and “residual waste” (excluding recyclables).
3. The waste volume in this study is based on the median values of “food waste”, “recyclables” and “residual waste”. The total amount of household waste is the sum of these three types, rather than the median and mean values directly from the table.
4. The data cover a period of 15 months, from May 2020 to July 2021, with some months missing. The “Count” line indicates the number of months for which data was used.

**Table 1:** Basic situation of household waste treatment facilities in each district in Beijing, 2021.

Number	Name of Facilities	Location	Main Processing Technology	Designed Processing Capacity/t/d
1	Gao'antun Waste Incineration Plant	Chaoyang District	Incineration	1600
2	Chaoyang Waste Incineration Center	Chaoyang District	Incineration	1800
3	Haidian District Circular Economy Industrial Park Renewable Energy Power Plant	Haidian District	Incineration	1800
4	Lujiashan Waste Incineration Plant	Mentougou District	Incineration	3000
5	Tongzhou District Renewable Energy Power Plant	Tongzhou District	Incineration	2250
6	Shunyi District Integrated Waste Treatment Plant	Shunyi District	Incineration	1140
7	Asuwai Waste Incineration Power Plant	Changping District	Incineration	3000
8	Nangong Domestic Waste Incineration Plant	Daxing District	Incineration	1000
9	Huairou District Domestic Waste Incineration Power Plant	Huairou District	Incineration	600
10	Pinggu District Integrated Waste Treatment Plant	Pinggu District	Incineration	300
11	Miyun Integrated Waste Treatment Center	Miyun District	Incineration	600
12	Chaoyang District Food Waste Treatment Plant	Chaoyang District	Biochemistry	400
13	Haidian Dagong Village Food Waste Treatment Plant	Haidian District	Biochemistry	400
14	Fengtai Food Waste Treatment Plant	Fengtai District	Biochemistry	500
15	Fengtai Wet-degradation Treatment Plant	Fengtai District	Biochemistry	600
16	Shougang Food Waste Treatment Plant	Mentougou District	Biochemistry	100
17	Yanshan Integrated Waste Treatment Plant	Fangshan District	Biochemistry	250
18	Dongcun Integrated Waste Treatment Plant	Tongzhou District	Biochemistry	700
19	Dongcun Food Waste Demonstration Project	Tongzhou District	Biochemistry	150
20	Organic Matter Ecological Treatment Station of Tongzhou District (Kitchen)	Tongzhou District	Biochemistry	200
21	Shunyi Integrated Waste Treatment Center (Kitchen)	Shunyi District	Biochemistry	100
22	Asuwei Integrated Waste Treatment Plant	Changping District	Biochemistry	1600
23	Nangong Waste Composting Plant	Daxing District	Biochemistry	2000
24	Nangong Food Waste Treatment Plant	Daxing District	Biochemistry	400
25	Wolujie Integrated Waste Treatment Plant	Huairou District	Biochemistry	200
26	Miyun Integrated Waste Treatment Center (Kitchen)	Miyun District	Biochemistry	30
27	Yanqing Integrated Waste Treatment Plant	Yanqing District	Biochemistry	350
28	Gao'antun Sanitary Landfill Plant	Chaoyang District	Landfill	1000
29	Liulitun Sanitary Landfill Plant	Haidian District	Landfill	1500
30	Fengtai Circular Economy Industrial Park of Waste Landfill Plant	Fengtai District	Landfill	2000
31	Zhaitang Sanitary Landfill Plant	Mentougou District	Landfill	41
32	Dongnanzhao Sanitary Landfill Plant	Fangshan District	Landfill	1000
33	Shunyi District Integrated Waste Treatment Plant	Shunyi District	Landfill	1140
34	Anding Sanitary Landfill Plant	Daxing District	Landfill	1400
35	Huairou District Sanitary Landfill Plant	Huairou District	Landfill	300
36	Xiaozhangjiakou Sanitary Landfill Plant	Yanqing District	Landfill	150
37	Yongning Sanitary Landfill Plant	Yanqing District	Landfill	100
38	Xiaowuki Waste Transfer Station	Chaoyang District	Transfer	2000
39	Datun Waste Transfer Station	Chaoyang District	Transfer	1800
40	Wuluju Waste Transfer Station	Haidian District	Transfer	1500
41	Majialou Waste Transfer Station	Fengtai District	Transfer	2000
42	Fengtai Circular Economy Industrial Park Pretreatment Screening Plant	Fengtai District	Transfer	2000
43	Yamenkou Waste Transfer Station	Shijingshan District	Transfer	500
44	Putaozui Waste Transfer Station	Mentougou District	Transfer	400
45	Chengguan Waste Transfer Station	Fangshan District	Transfer	200
46	Tongzhou District Waste Transfer Station	Tongzhou District	Transfer	800

Data source: Beijing Municipal Commission of Urban Management.

## 3 Methodology

### 3.1 Analytical framework

At present, LCA [2,5,20,31], Hybrid Life Cycle Assessment (HLCA) [32], Input-Output model [33], Pinch Analysis [6,16,34] are the main methods used to study the environmental benefits such as GHG emissions of household waste system. In recent years, LCA, as a common method to calculate GHG emissions, has attracted attention in the waste industry. Meanwhile, studies on GHG emissions from the waste industry have often combined material flow analysis (MFA) with LCA [21,35]. In addition, some scholars have also combined the embodied carbon/energy method with LCA to calculate the energy-saving and emission reduction potential of material recycling or waste management systems [36,37]. This study will integrate LCA, MFA, IPCC (The Intergovernmental Panel on Climate Change) guidelines, and the embodied carbon/energy method to examine the GHG emissions and mitigation potentials across various aspects of the waste classification system. The entire process, from community collection to waste treatment, will be evaluated using LCA. The MFA method will be utilized to analyze the distribution of waste quantities. GHG emissions resulting from treatment, transportation processes, and energy consumption from equipment will be calculated following the calculation methods outlined in the IPCC guidelines. In addition, the reduction of GHG emissions through the recycling of recyclable materials will be assessed using the embodied carbon/energy method. It is worth noting that this life cycle assessment is not comprehensive and does not encompass the impact of other pollutants; instead, it primarily focuses on carbon footprint research through the utilization of LCA.

**Fig. 3:**
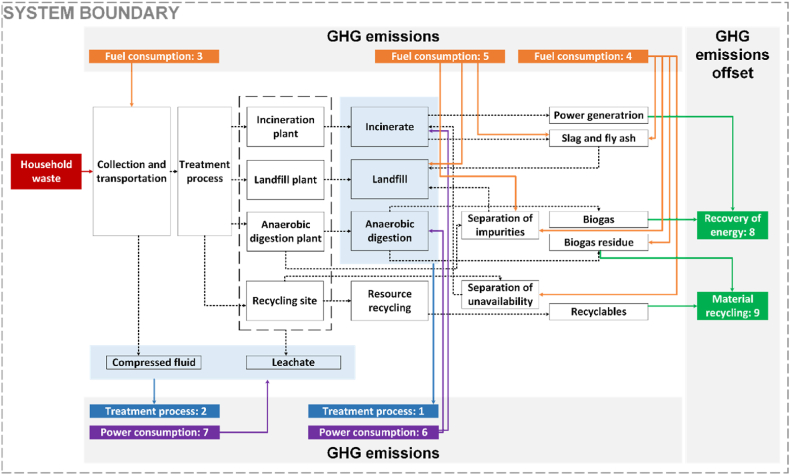
The household waste classification system.

The system is shown in Fig. 3, including the collection and transportation, treatment, and recyclables utilization. Household waste and energy (fuels and power) are regarded as the input items of the system, and GHG emissions and the offset are treated as the output items. The life cycle of pre-collection systems and vehicles is excluded, which is a topic worthy of further discussion. Given the data availability, obtaining specific information about transportation vehicles can be quite challenging, and that's why they are often overlooked in similar studies [38].

The objectives of the classification system include the following aspects: (1) quantify the GHG emissions and offsets for the entire life cycle of the waste classification system; (2) compare with the traditional mixed collection and transportation system; (3) predict the emission reduction potential of future waste classification systems. The functional unit is defined as the treatment of 1 t of household waste in Beijing.

### 3.2 Calculation method

The methodology and data for quantifying GHG emissions related to waste classification system are thoroughly described in this part. In this study, the GHG emissions (CO_2_, N_2_O, CH_4_) are all from the following three aspects (see Table 2): (1) GHG emissions from three treatment methods: incineration, landfill, and anaerobic digestion; (2) Fuel consumption including the landfill process and the vehicle transportation, etc.; (3) Power consumption including the power consumed from transfer stations and compression stations, power consumed by the incineration and anaerobic digestion plant, power consumed by the compression fluid and the leachate treatment. The GHG emissions offsets include recovery of energy and material recycling.

**Table 2:** Accounting links of GHG emissions and emissions offsets.

Links			Number	Incineration	Landfill	Anaerobic digestion	Recycling
GHG emissions	Disposal process	Treatment	1	✓	✓	✓	
		Compressed fluid and leachate	2	✓	✓	✓	✓
	Fuel consumption	Transport process before treatment	3	✓	✓	✓	✓
		Transport process after treatment	4	✓		✓	✓
		Use of landfill equipment	5	✓	✓	✓	
	Power consumption	Treatment	6	✓		✓	
		Compressed fluid and leachate	7	✓	✓	✓	✓
GHG emissions offsets	Recovery of energy		8	✓		✓	
	Material recycling		9			✓	✓

The calculation formula of Section 3.2 mainly references *IPCC 2006* [39]. The majority of the default factors for these calculations are also derived from *IPCC 2006*. The GWP values are obtained from *IPCC the Sixth Assessment Report (IPCC AR6) 2019*. Based on their 100-year global warming potential, the default coefficients are N_2_O (273 ± 130) and CH_4_-non fossil (27.0 ± 11), which are used to calculate the GHG equivalents of N_2_O and CH_4_.

#### 3.2.1 GHG emissions

##### 3.2.1.1 Treatment process

(1) Incineration

GHG from the incineration include CO_2_, CH_4,_ and N_2_O. In general, incineration produces more CO_2_ than CH_4_ and N_2_O [40]. In this study, CO_2_ emissions are calculated based on the components of the incinerated waste (such as paper/cardboard, and plastic, etc.), as outlined in Table 3. The calculation formulas presented in equations (1), (2).

(1) $$E_{inc.-{CO}_{2}}=\sum_{i}^{n}W_{i,1}\times {CF}_{i}\times {FCF}_{i}\times {OF}_{i}\times \frac{44}{12}\times {GWP}_{{CO}_{2}}$$

(2) $$W_{i,1}=\left(M_{1,1}\times {WF}_{i,1}+M_{1,2}\times {WF}_{i,2}\right)\times {dm}_{i}$$

**Table 3:** Data for estimating CO_2_ emissions from incineration based on household waste components.

Components	$M_{1}$/t/d	${WF}_{i,1}$/% [1]	${WF}_{i,2}$/% [41]	${dm}_{i}$/%	$W_{i,1}$/t/d	${CF}_{i}$/%	${FCF}_{i}$/%	${OF}_{i}$/%
Paper/Cardboard	12405.49 *M*_*1,1*_ = 9673.54 *M*_*1,2*_ = 2731.95	11.57	38.57	90	1955.66	46	1	95
Metal		2.75	**-**	100	266.02	**-**	**-**	
Glass		3.92	**-**	100	379.20	**-**	**-**	
Plastics		11.77	12.04	100	1467.43	75	100	
Textiles		4.18	2.64	80	381.26	50	20	
Rubber and Leather		1.6	3.57	84	211.91	67	20	
Wood/Bamboo		4.29	43.18	85	1355.44	50	**-**	
Food Waste		50.76	**-**	40	1964.12	38	**-**	
Others		0.92	**-**	90	80.10	3	100	

Note.

1M_1_ represents the total amount of residual waste and recyclables sent for incineration. In this study, the recyclables directed to the incineration plant are proportionally integrated into the corresponding components of the residual waste.

2. Recyclables can be categorized into five groups: Paper/Cardboard, Plastics, Textiles, Rubber and Leather, and Wood/Bamboo.

Data source.

Beijing Municipal Commission of Urban Management.

MoHURD, 2021; MoHURD, 2022; MoHURD, 2022b; NDRC, 2014.

Bian et al., 2022; Chen et al., 2020.

IPCC 2006; IPCC AR6 2019.

$E_{inc.-{CO}_{2}}$ = CO_2_-eq emissions (CO_2_) from waste incineration in the inventory year (t CO_2_-eq/d; t CO_2_-eq/yr);

$W_{i,1}$ = number of different components of household waste incinerated (dry matter) (t/d; t/yr);

${CF}_{i}$ = fraction of carbon in the dry matter (carbon content) (%);

${FCF}_{i}$ = fraction of fossil carbon in the total carbon (%);

${OF}_{i}$ = oxidation factor (%). ${OF}_{i}$ is 95 % [5];

44/12 = conversion factor from C to CO_2_;

i = components of the household waste incinerated, such as paper/cardboard, textiles, food waste, wood/bamboo, rubber and leather, plastics, metal, glass, and other inert waste;

${GWP}_{{CO}_{2}}$ = global warming potential of CO_2_. ${GWP}_{{CO}_{2}}$ is 1;

$M_{1,1}$ = total amount of residual waste as wet weight incinerated (t/d; t/yr);

$M_{1,2}$ = total amount of recyclables as wet weight incinerated (t/d; t/yr);

${WF}_{i,1}$ = proportion of components in the residual waste (as wet weight) (%);

${WF}_{i,2}$ = the proportion of components in the recyclables (as wet weight) (%);

${dm}_{i}$ = fraction of dry matter content in the component of the household waste incinerated (%).

*CF*_*i*_*, FCF*_*i*_*and dm*_*i*_ data are available in *IPCC 2006.*

N_2_O and CH_4_ in this study are calculated based on the amount of household waste incinerated and the default emission factors of N_2_O and CH_4_. These values are then converted into their respective global warming potential. The calculation formulas are provided in equations (3), (4).

(3) $$E_{inc.-N_{2}O}=M_{1}\times {EF}_{N_{2}O,1}\times 10^{-6}\times {GWP}_{N_{2}O}$$

(4) $$E_{inc.-CH_{4}}=M_{1}\times {EF}_{{CH}_{4},1}\times 10^{-6}\times {GWP}_{{CH}_{4}}$$

$E_{inc.-N_{2}O}$ = CO_2_-eq emissions (N_2_O) from waste incineration in the inventory year (t CO_2_-eq/d; t CO_2_-eq/yr);

$M_{1}$ = total amount of household waste as wet weight incinerated (t/d; t/yr);

${EF}_{N_{2}O,1}$ = N_2_O emission factor for household waste. Its value is 50 g N_2_O/t waste [39];

$10^{-6}$ = unit conversion factor;

${GWP}_{N_{2}O}$ = global warming potential of N_2_O. ${GWP}_{N_{2}O}$ is 273;

$E_{inc.-CH_{4}}$ = CO_2_-eq emission (CH_4_) from waste incineration in the inventory year (t CO_2_-eq/d; t CO_2_-eq/yr);

${EF}_{{CH}_{4},1}$ = aggregate CH_4_ emission factor. Its value is 0.2 kg CH_4_/Gg of waste [39];

${GWP}_{{CH}_{4}}$ = global warming potential of CH_4_. ${GWP}_{{CH}_{4},2}$ is 27.0.

(2) Landfill

GHG emissions from landfill mainly originate from CH_4_, which is produced by the degradation of organic matter under anaerobic conditions during the landfilling process. Decomposable portion of household waste encompasses food waste, wood/bamboo, paper/cardboard, etc. The data required for the calculations can be found in Table 4. The calculation formulas are presented in equations (5), (6), (7), (8), (9).

(5) $$E_{lan.-{CH}_{4}}=\sum_{i}^{n}{CH}_{4}\;{generated}_{i}\times \left(1-OX\right)\times {GWP}_{{CH}_{4}}$$

(6) $$\sum_{i}^{n}{CH}_{4}\;{generated}_{i}=\sum_{i}^{n}L_{o,i}$$

(7) $$L_{o,i}={DDOC}_{m,i}\times F\times 16/12$$

(8) $${DDOC}_{m,i}=W_{i,2}\times {MCF}_{1}\times DOC\times {DOC}_{f,i}$$

(9) $$W_{i,2}=M_{2}\times {WF}_{i,1}$$

**Table 4:** Data for estimating CH_4_ emissions from landfill based on household waste components.

Components	$M_{2}$/t/d	${MCF}_{1}$	DOC/%	${DOC}_{f,i}$	F	OX	${GWP}_{{CH}_{4}}$
Paper/Cardboard	2697.36	1.0	40	0.5	0.5	0.1	27
Metal			**-**				
Glass			**-**				
Plastics			**-**				
Textiles			24				
Rubber and Leather			39				
Wood/Bamboo			43				
Food Waste			15				
Others			**-**				

Note: 1. M_2_ encompasses two categories: residual waste sent to landfill plants and impurities from anaerobic digestion.

IPCC 2006; IPCC AR6 2019; NDRC, 2011.

$E_{lan.-{CH}_{4}}$ = CO_2_-eq emissions (CH_4_) from landfill in the inventory year (t CO_2_-eq/d; t CO_2_-eq/yr);

${CH}_{4}\;{generated}_{i}$ = CH_4_ generated from various decomposable matter (t CH_4_);

OX = oxidation factor, 0.1 [39];

${GWP}_{{CH}_{4}}$ = global warming potential of CH_4_, 27.0;

$L_{o,i}$ = CH_4_ generation potential (t CH_4_);

${DDOC}_{m,i}$ = mass of decomposable DOC (t);

F = fraction of CH_4_ in generated landfill gas, 0.5 [39];

${MCF}_{1}$ = CH_4_ correction factor for aerobic decomposition in the year of deposition, 1.0 [39];

DOC = degradable organic carbon in the year of deposition (kg C/kg waste). The default values can be found in *IPCC 2006*;

${DOC}_{f,i}$ = fraction of DOC that can decompose, 0.5 [39];

$W_{i,2}$ = number of different components of household waste treated by landfill (t/d; t/yr);

$M_{2}$ = total amount of household waste as wet weight landfilled (t/d; t/yr);

${WF}_{i,1}$ = proportion of components in the residual waste (as wet weight) (%). The data is presented in Table 3;

16/12 = molecular weight ratio CH_4_/C.

(3) Anaerobic digestion

Since the N_2_O emission factor for anaerobic digestion is assumed to be negligible [39], CH_4_ is considered the primary GHG emission in this section. The quantity of CH_4_ recovered is accounted for in the biogas power generation of the emissions offsets in Section 3.2.2. The CH_4_ emissions from the anaerobic digestion can be estimated by equation (10).

(10) $$E_{AD-CH_{4}}=M_{3}\times {EF}_{{CH}_{4},2}\times 10^{-3}\times {GWP}_{{CH}_{4}}$$

$E_{AD-CH_{4}}$ = CO_2_-eq emissions (CH_4_) from anaerobic digestion in the inventory year (t CO_2_-eq/d; t CO_2_-eq/yr);

$M_{3}$ = mass of organic waste treated by anaerobic digestion, 5165.10 t/d;

${EF}_{{CH}_{4},2}$ = emission factor for anaerobic digestion, wet weight, 1 g CH_4_/kg waste [39];

${GWP}_{{CH}_{4}}$ = global warming potential of CH_4_, 27.0.

(4) Compressed liquid and leachate

The primary GHG present in compressed fluid and leachate are CH_4_ and N_2_O. The compressed liquid is produced during compression. Leachate is produced during the stacking process, such as at the transfer stations, incineration plants, landfill sites, anaerobic digestion plants, and recycling facilities. The pertinent formulas for calculating GHG emissions (equations (11), (12), (13)) are listed.

(11) $$E_{wat.-CH_{4}}=V_{i}\times \left(TOW\times {EF}_{{CH}_{4},3}\right)\times 10^{-6}\times {GWP}_{{CH}_{4}}$$

(12) $${EF}_{{CH}_{4},3}=B_{o}\times {MCF}_{2}$$

(13) $$E_{wat.-N_{2}O}=V_{i}\times {TN}_{F}\times {EF}_{N_{2}O,2}\times 10^{-6}\times 44/28\times {GWP}_{N_{2}O}$$

$E_{wat.-CH_{4}}$ = CO_2_-eq emissions (CH_4_) from wastewater in the inventory year (t CO_2_-eq/d; t CO_2_-eq/yr);

$V_{i}$ = the volumes of compressed fluid and leachate (m^3^/d). The calculation parameters for the volumes of compressed fluid and leachate are provided in Table 5.

**Table 5:** The amount of compressed fluid and leachate from household waste.

	L/d
Compressed fluid	MSW*5.6 %
Leachate	0.38 × (MSW- MSW*5.6 %)÷30 × 7

TOW = total organics in wastewater (kg BOD/d). BOD is 11,035 mg/L [5];

${EF}_{{CH}_{4},3}$ = emission factor (kg CH_4_/kg BOD);

${GWP}_{{CH}_{4}}$ = global warming potential of CH_4_. ${GWP}_{{CH}_{4},2}$ is 27.0;

$B_{o}$ = maximum CH_4_-producing capacity, 0.6 kg CH_4_/kg BOD [39];

${MCF}_{2}$ = methane correction factor, 0.16 [5];

$E_{wat.-N_{2}O}$ = CO_2_-eq emissions (N_2_O) from wastewater in the inventory year (t CO_2_-eq/d; t CO_2_-eq/yr);

${TN}_{F}$ = the total amount of nitrogen (mg/L). TN is 500 mg/L [5];

${EF}_{N_{2}O,2}$ = emission factor for N_2_O emissions from wastewater, 0.005 kg N_2_O–N/kg N [39];

${GWP}_{N_{2}O}$ = global warming potential of N_2_O, 273;

44/28 = conversion of kg N_2_O–N into kg N_2_O.

##### 3.2.1.2 Fuel consumption

The fuel consumption mainly comes from three aspects: (1) fuel consumption of vehicles during the waste collection and transportation; (2) transportation of incineration residues and fly ash, impurities from anaerobic digestion, biogas residues from anaerobic digestion, and recyclables which are not recycled; (3) the use of landfill equipment. The fuel is mainly diesel. The residues, fly ash, impurities, and biogas residues accounted for 10 %, 10 %, 10 %, and 20 % of the wet waste volume, respectively [5,29]. The fuel consumption of vehicles during the collection and transportation is calculated in equation (14).

(14) $$C_{fuel-trans.}=D_{i}\times C_{empty}+D_{i}\times C_{full}$$

$C_{fuel-trans.}$ = fuel consumption of vehicles during waste collection and transportation (L/d);

$D_{i}$ = average distance from district centers to different treatment facilities (km);

$C_{empty}$ = fuel consumption of a transport vehicle when unloaded (L/100 km);

$C_{full}$ = fuel consumption of a transport vehicle when fully loaded (L/100 km).

The average distance from district centers to different treatment facilities is calculated based on the average distance between the 16 district centers and various treatment facilities. These data can be obtained from the Amap. The average distances from the center of each district to the incineration, landfill, anaerobic digestion, and recycling station, as well as the average distances from the center of each district to the transfer station, and the transfer station to the four treatment facilities are 60, 71, 59, 63, 49 and 14 km, respectively. The vehicles exhibit a fuel consumption rate of 8 L/100 km when they are not carrying a load, and 11 L/100 km when they are fully loaded [29].

The fuel consumption during the landfill process is 6.72 MJ/ton [5]. The calorific value of diesel is 0.038 GJ/L [42]. The basic data for the total fuel consumption during vehicle transportation are shown in Table 6 The calculation of GHG emissions can be expressed by equation (15).

(15) $$E_{fuel,i}=C_{i}\times {EF}_{fuel}\times {GWP}_{{CO}_{2}}$$

**Table 6:** Basic data of vehicle transportation and total fuel consumption.

		Transport times	Total distance /100 km	Total fuel consumption /L
Incineration	Before transfer station	1386	679.09	12902.67
	After transfer station		194.03	3686.48
	Direct transport		831.54	15799.19
Landfill	Before transfer station	304	149.07	2832.29
	After transfer station		42.59	809.23
	Direct transport		216.00	4103.94
Anaerobic digestion	Before transfer station	822	402.88	7654.74
	After transfer station		115.11	2187.07
	Direct transport		485.10	9216.93
Recycling	Before transfer station	602	295.05	5606.01
	After transfer station		84.30	1601.72
	Direct transport		379.35	7207.72

Note: The fully loaded weight of the vehicles is 3490 kg [29].

$E_{fuel,i}$ = CO_2_-eq emissions from different aspects of fuel consumption (t CO_2_-eq/d; t CO_2_-eq/yr);

$C_{i}$ = number of different aspects of fuel consumption (t/d; t/yr);

${EF}_{fuel}$ = the carbon emission factor of diesel. The carbon emission factor of diesel is 2.63 kg/L [29];

i = different aspects of fuel consumption.

##### 3.2.1.3 Power consumption

The power consumption mainly comes from four aspects: (1) the equipment of the incineration plant. After the power generation, 30 % of the electricity will be returned to the plants [5]; (2) the equipment of the crushing and pulping process in the anaerobic digestion, which is 32 kWh/t waste [43]; (3) the equipment of compression in transport station, which is 3.3 kWh/t waste [5]; (4) the treatment of compressed liquid and leachate, which is 0.13 kWh/m^3^ wastewater [5,44]. The calculation of GHG emissions can be expressed by equation (16).

(16) $$E_{elec.-i}=P_{t,i}\times {EF}_{elec.}\times {GWP}_{{CO}_{2}}$$

$E_{elec.-i}$ = CO_2_-eq from different aspects of power consumption (t CO_2_-eq/d; t CO_2_-eq/yr);

$P_{t,i}$ = the number of different aspects of power consumption (kWh/d);

i = different aspects of power consumption;

${EF}_{elec.}$ = electricity grid emission factor, 0.5810 kg CO_2_/kWh [45].

#### 3.2.2 Avoided GHG emissions burdens

##### 3.2.2.1 Recovery of energy

(1) Incineration

Energy recovery involves household waste incineration for power generation. In this study, the heat energy generated during the incineration is first calculated based on the waste components. Subsequently, power generation is obtained using the thermoelectric conversion coefficient and power generation recovery efficiency. GHG emissions offsets need to be calculated based on electricity grid emission factor. The lower heating value takes into account the effects of humidity and hydrogen content, thus being derived by subtracting these influences from the higher heating value. The calculation formula for incineration power generation is presented in equations is presented in equation (17).

(17) $$E_{re.-power}=\sum_{i}^{n}W_{i,1}\times \frac{{HHV}_{i}-2594\times \left(9H_{i}+W_{w,i}\right)}{3600kJ/kWh}\times {EF}_{efficiency}\times {EF}_{elec.}\times {GWP}_{{CO}_{2}}$$

$E_{re.-power}$ = GHG emissions offsets from incineration power generation (t CO_2_-eq/d; t CO_2_-eq/yr);

$W_{i,1}$ = weight of different components of household waste incinerated (t/d; t/yr);

${EF}_{efficiency}$ = power generation recovery efficiency (%), 22.05 % [27,46];

${HHV}_{i}$ = higher heating value of material *i*, dry matter (kJ/kg);

$H_{i}$ = content of element *H* in material *i*, dry matter (%);

$W_{w,i}$ = amount of moisture in waste *i* incinerated, dry matter (%).

The values and sources of *HHV*_*i*_, *H*_*i*_ and *W*_*w,i*_ can be found in Table 7.

(2) Anaerobic digestion

**Table 7:** The average higher heating value (HHV), hydrogen (H) element content, and moisture content of various components in household waste.

Components	${HHV}_{i}$/kJ/kg	$H_{i}$/%	$W_{w,i}$/%
Paper/Cardboard	15,894	6.01	29.23
Metal	**-**	**-**	1.30
Glass	**-**	**-**	4.54
Plastics	43,448	0.73	25.69
Textiles	20,162	38.09	29.67
Rubber and Leather	29,789	4.31	25.69
Wood/Bamboo	19,464	40.5	31.59
Food Waste	15,386	7.04	68.18
Others	–		19.87

The end products of anaerobic digestion include biogas, biogas residues, and leachate. Biogas is mainly composed of CH_4_ (main component), CO, CO_2_, and H_2_S. Considering potential leakage ranging from 0 % to 10 % during anaerobic digestion, it is assumed that 95 % of the generated methane can ultimately be utilized for recovery and power generation [39]. Assuming that the power generation efficiency is 50 % [47,48], the GHG offsets achieved by the biogas power generation can be calculated using equation (18) [5,48].

(18) $$E_{re.-biogas}=\left(M_{AD-CH_{4}}\times 95\%\times 50100/3600\times 50\%\times {EF}_{elec.}-2.75\times M_{AD-CH_{4}}\right)\times {GWP}_{{CO}_{2}}$$

$E_{\text{re}.-\text{biogas}}$ = using biogas to generate electricity achieves offsets in GHG emissions compared to releasing methane (CH_4_) into the atmosphere (t CO_2_-eq/d; t CO_2_-eq/yr);

$M_{\text{AD}-CH_{4}}$ = $E_{\text{AD}-CH_{4}}/{\text{GWP}}_{CH_{4},2}$. It means the mass of available CH_4_ from anaerobic digestion for recovery (t CH_4_);

$2.75\times M_{AD-CH_{4}}$ = CO_2_-eq emissions from methane combustion. The CO_2_-eq emissions per unit of methane combustion are approximately 2.75.

##### 3.2.2.2 Material recycling

(1) Recyclables

Recycling of recyclables has the potential to replace certain production processes, thereby reducing energy consumption. The proportions of various recyclable materials are outlined in Table 3. In this case, the embodied energy/carbon method is utilized to calculate the GHG emissions offsets [37,49], as demonstrated in equation (19). Table 8 presents the embodied carbon coefficients for both raw materials and recyclables.

(19) $$E_{re.-recyclables}=\sum_{i}^{n}M_{raw,i}\times f_{raw,i}-\sum_{i}^{n}M_{recy.,i}\times f_{recy.,i}$$

**Table 8:** Embodied carbon coefficients of raw materials and recyclables (kg CO_2_/kg).

	Raw materials	Recyclables
Paper/Cardboard	1.89	1.36
Waste plastic	2.28	0.46
Glass bottles	1.02	0.38
Rubber	3.18	–
Textile	6.71	–

Note: 1. The data for Paper/Cardboard, Waste plastic, Glass bottles, and Rubber are sourced from ICE Version 1.6a (2008). The data for textile is sourced from Semba et al. (2020).

$E_{re.-recyclable}$ = GHG emissions offsets from recyclable recycling (t CO_2_-eq/d; t CO_2_-eq/yr);

$M_{raw,i}$ = number of raw materials (t/d; t/yr);

$M_{recy.,i}$ = number of recyclables (t/d; t/yr);

$f_{raw,i}$ = embodied carbon coefficients of raw materials (kg CO_2_-eq/kg);

$f_{recy.,i}$ = embodied carbon coefficients of recyclables (kg CO_2_-eq/kg).

(2) Biogas residues

One of the significant methods for recycling anaerobic biogas residues involves its transformation into organic fertilizer through composting, enabling the reduction of carbon emissions. Taking into account the carbon transfer and storage during the composting process, the net carbon flux in this study is −0.055 t CE/t waste [5,50]. The calculation method is depicted in equation (20).

(20) $$E_{re.-bio.}=M_{4}\times 20\%\times {EF}_{bio.}\times \frac{44}{12}$$

$E_{re.-bio.}$ = GHG emissions offsets from biogas residues after anaerobic digestion (t CO_2_-eq/d; t CO_2_-eq/yr);

$M_{4}$ = weight of organic waste treated by anaerobic digestion (t/d; t/yr);

${EF}_{bio.}$ = net carbon flux.

### 3.3 Scenario design

To analyze the emission reduction potential under different measures, three scenarios including the BAU Scenario, the Classification Efficiency Scenario, and the Comprehensive Scenario are set up to explore the emission trajectories of the waste classification system until 2060. Firstly, in the BAU Scenario, the parameters such as annual classification efficiency are the same as the base year 2021. In this scenario, changes in future waste volumes are projected to estimate the associated GHG emissions and emissions offsets under future waste classification. Secondly, in the Classification Efficiency Scenario, the classification rate will continue to increase, and it will affect the proportion of waste in different treatment methods and the proportion of each component in residual waste. Thirdly, the Comprehensive Scenario further incorporates energy technologies.

#### 3.3.1 BAU scenario

In the BAU Scenario, the waste growth rate is the key variable, and the classification efficiency and energy technologies improvements are not considered. In addition to the increase in waste volume, the classification efficiency and the energy efficiency of each inventory year are the same as the base year 2021, and other coefficients such as emission factors are consistent. Waste quantity prediction referenced approaches from similar research [51,52] and established a multiple linear regression model with gross domestic product (GDP), population, and electricity consumption as predictive variables to forecast household waste generation in Beijing up to 2060. The model details are presented in Table 9 and Table 10.

**Table 9:** Parameters of the household waste prediction model.

Model Summary					
Multiple -regression model	R	R square	Adjusted R-squared	Standard Deviation	Durbin-Waston
	.942^a^	0.887	0.881	88.9185	0.530
a. Independent variable variables: (Intercept), Electricity consumption, GDP, population					
b. Dependent variable: waste removal volume

**Table 10:** Results of the household waste prediction model.

Coefficient							
model		Unstandardized coefficient		Standardized Coefficient	*t*-test	significance	Multicollinearity test
		B	Standard Deviation	Beta			VIF
Multiple-regression model	Intercept	−310.328	61.127		−5.077	.000	
	population	.629	.059	1.154	10.657	.000	5.203
	GDP	.010	.002	.417	4.311	.000	4.155
	Electricity consumption	−.500	.090	−.670	−5.544	.000	6.487
a. Dependent variable: waste removal volume							

#### 3.3.2 Classification Efficiency Scenario

In addition to the waste growth rate, classification efficiency is another key variable in the waste classification system, and it is also an important variable that mainly affects waste volume for terminal treatment. It affects the waste volume of transportation, the amount of terminal treatment, and the proportion of waste components.

Based on the waste components in 2021, it is found that the maximum classification rates of food waste and recyclables are about 54 % and 37 %, respectively. It is expected that the waste classification rate will increase apace in the short term, and waste classification work will be easy to advance. In the long term, the growth rate of the waste classification will slow down. The classification efficiency is shown in Table 11.

**Table 11:** Classification rate of the food waste, recyclables and residual waste from 2021 to 2060.

	2021	2030	2040	2050	2060
Food waste	26.0 %	36.0 %	46.0 %	50.0 %	54.0 %
Recyclables	19.0 %	25.0 %	31.0 %	34.0 %	37.0 %
Residual waste	55.0 %	39.0 %	23.0 %	16.0 %	9.0 %

Note: Based on the waste quantity and composition from 2021, calculate the maximum separation rate for kitchen waste and recyclable materials.

The proportion of waste components corresponding to different classification efficiencies can be seen in Table 12 and Table 13. The proportion of the treatment methods corresponding to different classification efficiencies can be seen in Table 14.

**Table 12:** Components of residual waste at different classification efficiencies.

	2021	2030	2040	2050	2060
Paper/Cardboard	11.57 %	10.99 %	9.60 %	7.31 %	1.44 %
Metal	2.75 %	3.88 %	6.58 %	9.45 %	16.81 %
Glass	3.92 %	5.53 %	9.37 %	13.48 %	23.96 %
Plastics	11.77 %	11.18 %	9.77 %	7.43 %	1.47 %
Textiles	4.18 %	3.97 %	3.47 %	2.64 %	0.52 %
Rubber And Leather	1.6 %	1.52 %	1.33 %	1.01 %	0.20 %
Wood/Bamboo	4.29 %	4.07 %	3.56 %	2.71 %	0.54 %
Food Waste	50.76 %	45.94 %	34.43 %	24.49 %	−0.91 %
Other	0.92 %	1.30 %	2.20 %	3.16 %	5.62 %

Note: Due to the improved efficiency of waste classification, there have been changes in the composition of residue waste. This portion is calculated based on the information in Table 10 and the projected amount of waste.

**Table 13:** Components of recyclables at different classification efficiencies.

	2021	2030	2040	2050	2060
Paper/Cardboard	38.57 %	37.62 %	37.05 %	36.83 %	36.65 %
Plastics	12.04 %	17.60 %	21.01 %	22.27 %	23.32 %
Textiles	2.64 %	5.01 %	6.46 %	7.00 %	7.44 %
Rubber And Leather	3.57 %	3.86 %	4.04 %	4.11 %	4.16 %
Wood/Bamboo	43.18 %	35.90 %	31.44 %	29.79 %	28.42 %

Note: The increased efficiency in waste classification has led to changes in the composition of recyclables. This data is calculated based on the data from Table 10 and the projected amount of waste.

**Table 14:** The proportion of treatment methods at different classification efficiencies.

	2021	2030	2040	2050	2060
Incineration	44.5 %	32.0 %	18.9 %	13.1 %	7.4 %
Landfill	9.8 %	7.0 %	4.1 %	2.9 %	1.6 %
Anaerobic digestion	26.4 %	36.0 %	46.0 %	50.0 %	54.0 %
Recycle	19.3 %	25.0 %	31.0 %	34.0 %	37.0 %

Note: Due to the enhanced efficiency of waste classification, the proportions of waste treated by different methods have undergone changes. This portion is calculated using the data from Table 10 and the projected amount of waste.

#### 3.3.3 Comprehensive Scenario

According to the different characteristics of the technologies and their different positions in the waste classification system, the progress of energy technologies under the Comprehensive Scenario can be divided into three different categories: the improvements of existing energy utilization technologies, the substitution of energy technologies, and the application of untapped energy technologies.

The first category is the improvements of existing energy utilization technologies, including improving the recycling rate of recyclables; improving the recovery efficiency of incineration power generation, and improving the conversion efficiency of biogas power generation. The parameters of the energy technologies are shown in Table 15.

**Table 15:** Parameters of different energy technologies.

Category	Specific aspects	2021	2030	2040	2050	2060
1	the recycling rate of recyclables	35.00 %	51.25 %	67.50 %	83.75 %	100.00 %
	the recovery efficiency of incineration power generation	22.05 %	26.55 %	31.05 %	35.55 %	40.05 %
	the conversion efficiency of biogas power generation	50.00 %	55.00 %	60.00 %	65.00 %	70.00 %
2	the emission factor of transportation (before treatment)	2.63	2.38	2.13	1.87	1.62
	the emission factor of transportation (after treatment)	2.63	2.37	2.11	1.85	1.59
3	Power generation efficiency of the ICE used for LFG	0.00 %	40.00 %	45.00 %	50.00 %	55.00 %
	the efficiency of gas collection from the landfills	0.00 %	70.00 %	75.00 %	80.00 %	85.00 %
	the recovery rate of incinerated slag	0.00 %	25.00 %	30.00 %	35.00 %	40.00 %

Note.

1. The recycling rate of recyclables: Based on the scenario for increasing recycling rates set by Dong et al., 2018 (assuming all recyclables can be recycled), this study establishes a target vision of achieving a 100 % recycling rate by the year 2060 for long-term development.

*2. The recovery efficiency of incineration power generation: According to the latest European policies, waste incineration facilities installed after 2009 should achieve an energy recovery rate of at least 65 %* [53]*. Hence, this study establishes an incineration energy recovery rate of 40.05 %, which falls within the feasible scope of the long-term development goal*.

*3. The conversion efficiency of biogas power generation: A comparative review of biogas generation technologies has indicated that thermal efficiency can be enhanced up to 90 % through cogeneration* [54]*. Therefore, the calculations in this study assume 70 % as the technical limit for energy recovery from biogas generation*.

4. The emission factor of transportation (before treatment and after treatment): The current CO2 emission factor for diesel is 2.63 kg/L. In theory, electric vehicles can fully replace diesel vehicles for waste transportation. Therefore, this study set the proportions of electric vehicles replacement for diesel vehicles at 0 %, 15 %, 30 %, 45 %, and 60 % for the years 2021, 2030, 2040, 2050, and 2060 respectively. The CO2 emission factor of waste transportation was then calculated using the technological penetration formula presented in Section 3.3.3.

*5. Power generation efficiency of the ICE used for LFG and the efficiency of gas collection from the landfills: Currently, the internal combustion engine (ICE) used for landfill gas (LFG) utilization has a power generation efficiency of about 40 %. Therefore, this study sets a long-term development goal for ICE's power generation efficiency with LFG at 55 % by 2060* [55]. *According to data from a survey of gas collection systems at 23 landfills in Denmark, the gas collection efficiency of these landfills reached up to 86 %, with an average of 50 %* [56]*. Therefore, this study establishes a long-term development goal of 85 % for landfill gas collection efficiency by 2060*.

6. The recovery rate of incinerated slag: After reviewing the literature, no specific data is available to determine the percentage of incineration slag that is converted into building materials. Due to this lack of data, the parameters of this study were established by referencing the recyclable percentage of sustainable building materials from Sahlol et al., 2021.

From the perspective of energy technologies substitution, the fuel of transshipment vehicles will switch from diesel to electricity. With the reduction of diesel vehicles and the increase of electric vehicles, the penetration rate of electricity technology will gradually increase, affecting the emission factors of transportation. The transportation emission factors influenced by technological penetration are calculated as shown in equations (21), (22) (referring to Ref. [57]. The parameters are presented in Table 15.

(21) $${EF}_{i,1}={EF}_{i0}\times \left(1-T\right)+\left(2\times \frac{15.5}{19}\right)\times {EF}_{elec.}\times T$$

(22) $${EF}_{i,2}={EF}_{i0}\times \left(1-T\right)+\left(\frac{15.5}{10}\right)\times {EF}_{elec.}\times T$$

${EF}_{i,1}$ = emission factor of transportation (transport process before treatment) (kg/L);

${EF}_{i0}$ = carbon emission factor of diesel (kg/L), 2.63 kg/L [29];

T = technology penetration rate;

$\left(2\times \frac{15.5}{19}\right)$ = conversion coefficient. This coefficient is calculated based on data obtained from China's automotive energy consumption.

${EF}_{i,2}$ = emission factor of transportation (transport process after treatment) (kg/L);

$\left(\frac{15.5}{10}\right)$ = conversion coefficient. This coefficient is calculated based on data obtained from China's automotive energy consumption.

The energy technologies in the future include recovering landfill gas to generate electricity and converting incinerated slag into building materials. The parameters are shown in Table 15. The formula and parameters for calculating landfill gas recovery electricity generation can be found in equation (23) (referring to Ref. [58]). The method to calculate the emissions offsets of the slag is given in Section 3.2.2.

(23) $$E_{re.-{lan.CH}_{4}}={LHV}_{{CH}_{4}}\times M_{lan.-{CH}_{4}}\times 1000\times \eta_{ICE}\times \eta_{CL}/3600\times {EF}_{elec.}\times {GWP}_{{CO}_{2}}$$

$E_{re.-{lan.CH}_{4}}$ = GHG emissions offsets from landfill gas recovery (t CO_2_-eq/d; t CO_2_-eq/yr);

${LHV}_{{CH}_{4}}$ = low calorific value of methane (MJ/kg), 51.88 MJ/kg CH_4_ [58];

$M_{lan.-{CH}_{4}}$ = mass of available CH_4_ from landfill for recovery in the inventory year (t CH_4_);

$\eta_{ICE}$ = generation efficiency (%), 55 % [58];

$\eta_{CL}$ = collection efficiency (%), 85 % [58].

## 4 Results

### 4.1 GHG emission reduction benefits from waste classification

Based on the statistical data from 11 months after the new round of waste classification in Beijing, we have calculated the GHG emissions of the classification system and compared its results with those of the traditional mixed collection and transportation system to determine whether waste classification has a positive impact on GHG emissions. The calculation includes three parts: the collection and transportation, the treatment processes, and the resource recovery. In addition, we have factored in the potential emission reduction through energy recovery and material recycling.

#### 4.1.1 Avoid GHG burdens in the classification system

**Fig. 4:**
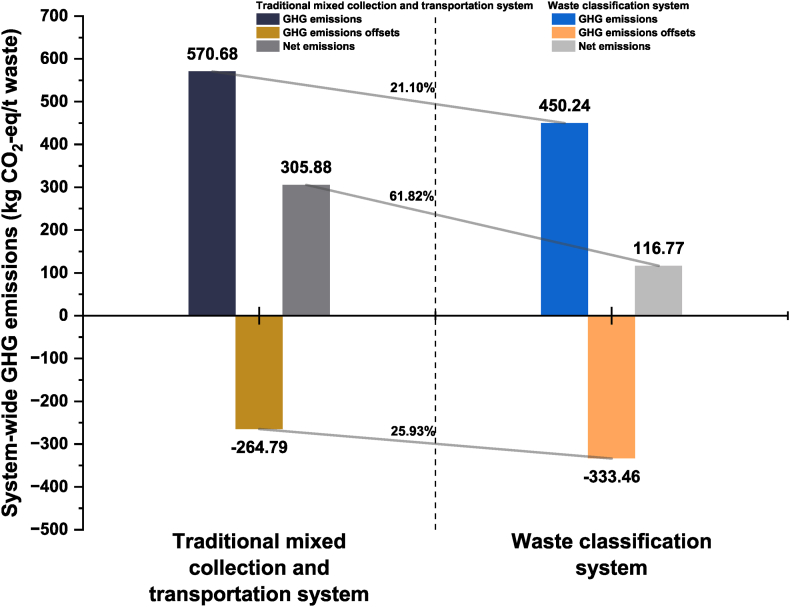
Comparison of system-wide GHG emissions between the waste classification system and the traditional mixed collection and transportation system in 2021.

Fig. 4 shows the comparison of system-wide GHG emissions between the two systems in 2021. It can be seen that the net emissions from the waste classification system are 116.77 kg CO_2_-eq/t waste, which is 61.82 % less than that of the traditional mixed collection and transportation system by 189.11 kg CO_2_-eq/t waste. This is due to a 120.44 kg CO_2_-eq/t waste reduction in GHG emissions from classification system and a 68.67 kg CO_2_-eq/t waste increase in GHG offsets. This shows that improvements in system-wide GHG emissions of waste classification systems not only reduce GHG emissions but also increase the number of GHG offsets.

#### 4.1.2 GHG in each link of classification system

**Fig. 5:**
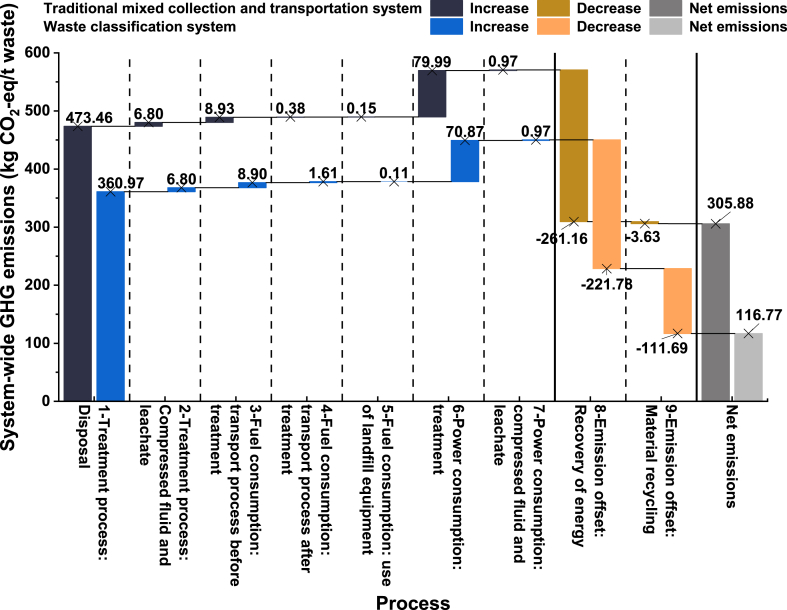
Each link's contribution to net emissions.

According to the data presented in Fig. 5, the emission reduction potential of the two systems differs in terms of GHG emissions and GHG emissions offsets.

For Links 1 to 7, the GHG emissions of the two systems are different, and therefore the emission potential of the two systems in these links is also different. Specifically, the GHG emissions of the traditional mixed collection and transportation system in these links are higher than those of the waste classification system, so its emission potential is also higher. Link 1 is the main contributor to the GHG emissions at 361–473 kg CO_2_-eq/t waste. In terms of emission reduction potential, Link 1 also has a higher emission reduction potential. The waste classification system reduces about 112.49 kg CO_2_-eq/t waste in this link, which is much higher than other links. Emissions in Link 1 are related to waste treatment methods, so the emission reduction potential of the waste classification system from GHG emissions mainly depends on the waste flow and treatment strategies.

For Links 8 to 9, there are also some differences in the emission reduction potential of the two systems in terms of GHG offsets. Specifically, the waste classification system has a higher potential for emission reduction in these links, as the total GHG offsets in these links are higher. In these links, the waste classification system can obtain a higher amount of GHG offsets through material recycling. The amount of GHG offsets by material recycling is about 30 times that of the traditional mixed collection and transportation system, increasing 108.06 kg CO_2_-eq/t waste. However, the energy recovery from incineration power generation may be limited.

In different links, the emission potential and emission reduction potential of the two systems are different. When formulating emission reduction strategies, it is necessary to take different emission reduction measures for different links and treatment methods to maximize the emission reduction potential of the waste classification system.

#### 4.1.3 Avoid GHG burdens in each treatment method of classification system

**Fig. 6:**
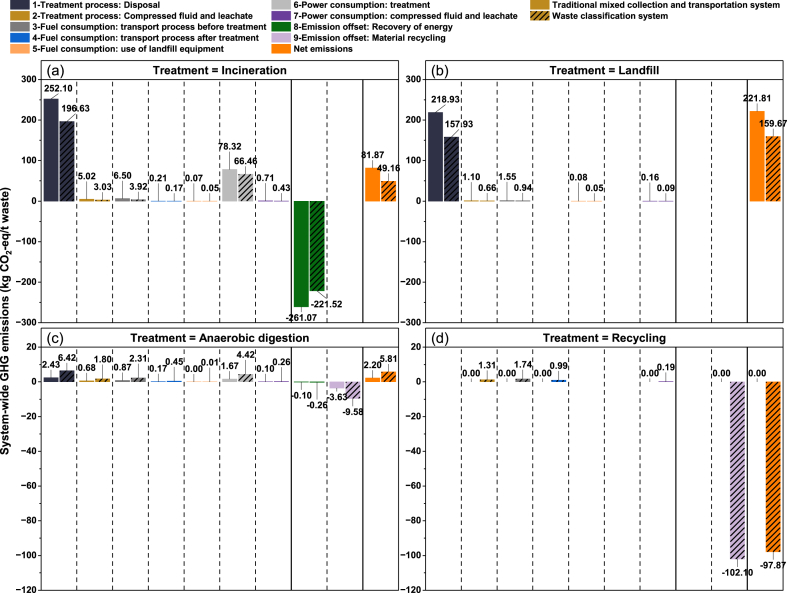
Each treatment method's GHG in two systems.

To compare the differences between GHG emissions and offsets of the four treatments and determine the emission reduction potential of the four treatments, the system-wide GHG emissions of each link in Fig. 5 are divided into four items, namely, incineration, landfill, anaerobic digestion, and recycling (see Fig. 6).

Fig. 6 shows the GHG emissions, GHG offsets, and net emissions of the four treatment methods in each link of the two systems.

In terms of GHG emissions, both incineration, and landfill have significant GHG emissions, ranging from 160 to 343 kg CO_2_-eq/t waste. The emissions from anaerobic digestion and recycling are relatively low, mostly below 20 kg CO_2_-eq/t waste.

In terms of GHG offsets, both incineration and recycling have significant offsets of −221.52 kg CO_2_-eq/t waste and −102.10 kg CO_2_-eq/t waste respectively, making a significant contribution to the net emissions of classification system. This is followed by anaerobic digestion, which is −9.84 kg CO_2_-eq/t waste. Whereas the landfill has no GHG offsets.

In terms of net emissions, the net emissions from incineration have decreased, but both the emissions and the offsets are relatively high. The net emissions from the landfill have also decreased. It has slightly lower emissions than incineration but has no GHG offsets. The net emissions from anaerobic digestion are 2.2–5.8 kg CO_2_-eq/t waste. Recycling has the lowest net emissions. It is essentially zero emissions and has higher offsets. This underscores the importance of energy recovery and material recycling to reduce net emissions.

In addition, comparing the four treatments, we speculate that recycling may have the highest emission reduction potential, followed by incineration. The anaerobic digestion and landfill have the lowest emission reduction potential.

Comparing the two systems, the net emissions of the waste classification system are lower than those of the traditional mixed collection and transportation system in three treatment methods. The net emissions of anaerobic digestion in the waste classification system are slightly higher than the traditional collection system, but the net emission intensity of anaerobic digestion is lower than that of landfill and incineration. Generally speaking, the two treatment methods of anaerobic digestion and recycling are the best in the waste classification system, followed by incineration, and landfill should gradually shift to the other three treatment methods.

In general, the existing waste classification system in Beijing provides 62 % more net emissions benefits than the traditional mixed collection and transportation system. Waste classification reduces GHG emissions (120.44 kg CO_2_-eq/t waste) and increases GHG offsets (−68.67 kg CO_2_-eq/t waste). Waste treated by incineration and landfill decreases by 3637.89 t waste/d and 1041.75 t waste/d, corresponding to an emission reduction of 711.08 t CO_2_-eq/d and 1350.89 t CO_2_-eq/d. Waste treated by anaerobic digestion and recycling increases by 3208.59 t waste/d and 1471.05 t waste/d, corresponding to an emission increase of 78.46 t CO_2_/d and an emission reduction of 2127.53 t CO_2_/d.

### 4.2 GHG emissions reduction potential from household waste classification

#### 4.2.1 Total GHG emissions of different scenarios

**Fig. 7:**
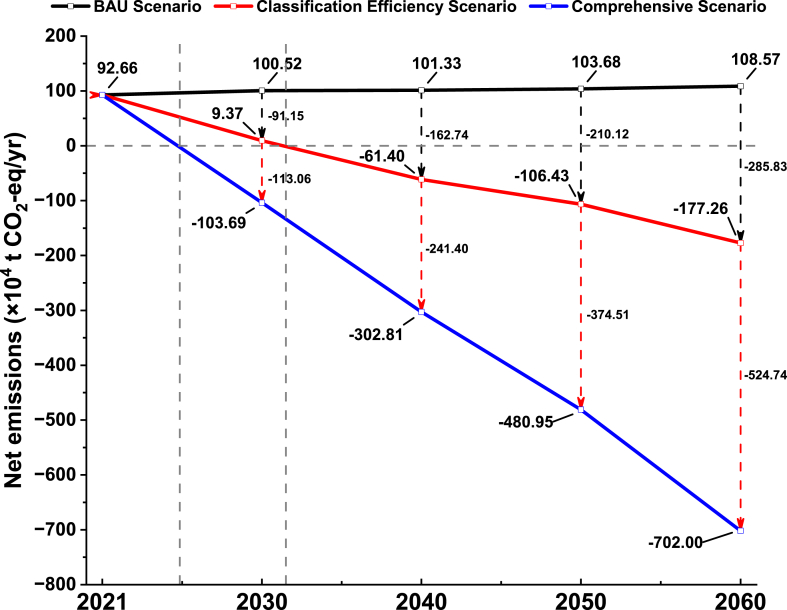
Net emissions in three scenarios from 2021 to 2060.

Three scenarios, namely the BAU Scenario, the Classification Efficiency Scenario, and the Comprehensive Scenario, are developed in this study to explore the emission trajectories of the waste classification system to 2060. Fig. 7 shows the net emissions from 2021 to 2060 under three scenarios of China's waste classification system.

In the BAU Scenario, according to the multivariate regression model, it is anticipated that waste generation will gradually and slowly increase from 2021 to 2060, reaching 929.46 × 10^4^ t/yr in 2060. During this period, the average growth rate of net emissions is approximately 4 %. By 2060, the net GHG emissions are projected to reach 108.57 × 10^4^ t CO_2_-eq/yr, which is 1.17 times the 2021 emissions.

In the Classification Efficiency Scenario, net-zero emissions will be achieved before 2035. Compared with the BAU Scenario, the Classification Efficiency Scenario has a significant and sustained reduction in net emissions from 2030 to 2060 and a significant increase in emission reduction. By 2060, the emissions will be reduced to −177.26 × 10^4^ t CO_2_-eq/yr, representing a reduction of 285.83 × 10^4^ t CO_2_-eq/yr relative to the BAU Scenario.

In the Comprehensive Scenario, net-zero emissions can be achieved earlier than in the Classification Efficiency Scenario and a larger range of net emission reduction can be achieved through further emission reduction measures. By 2060, the emissions will be further reduced to −702.00 × 10^4^ t CO_2_-eq/yr, representing a net emissions reduction of 524.74 × 10^4^ t CO_2_-eq/yr compared to the Classification Efficiency Scenario.

Overall, there is a large difference in net emissions between the Classification Efficiency Scenario and the Comprehensive Scenario, and their emission reduction effects are significant. Over time, emission reduction measures such as improving waste classification efficiency and increasing recycling rate have produced different degrees of emission reduction benefits, so the overall net emissions of the waste classification system are showing a continuous decreasing trend. Since the emission reduction measures adopted in each scenario are different, it is necessary to analyze the emission reduction potential of the emission reduction measures in each scenario to determine the optimal emission reduction strategy.

#### 4.2.2 Reduction potential of different measures

**Fig. 8:**
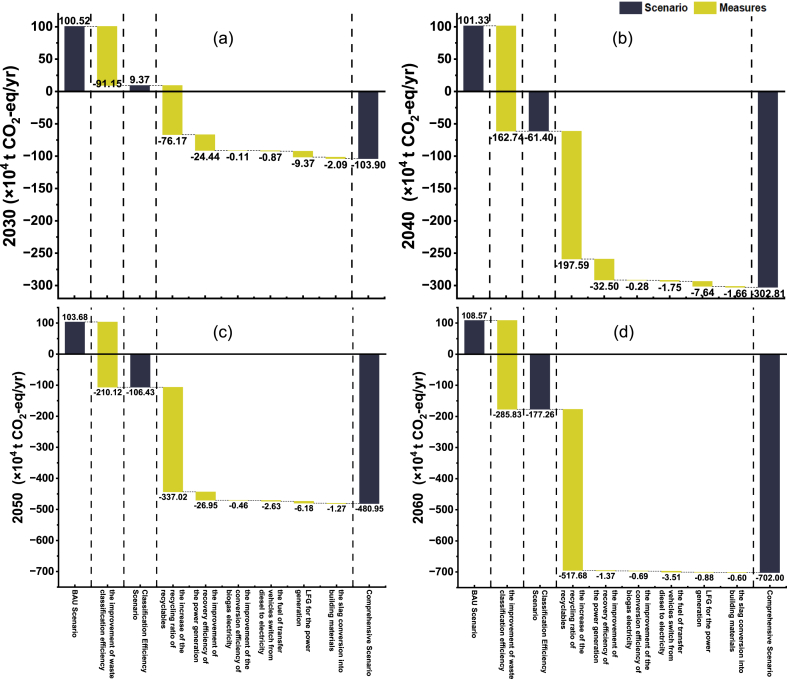
GHG emissions reduction potential of various measures from 2030 to 2060.

In the scenario setting, a total of seven different measures are set up to identify the emission reduction potential of different measures in the waste classification system. The result is shown in Fig. 8.

During the whole study period, the improvements in waste classification efficiency and the increase in recycling rate are the most important measures to promote emission reduction in the waste classification system. Its emission reduction increases from −167.32 × 10^4^ CO_2_-eq/yr in 2030 to −803.51 × 10^4^ CO_2_-eq/yr in 2060, accounting for 82 %–99 % of the overall emission reduction.

Improving the efficiency of incineration power generation and the collection and utilization of landfill gas are the secondary contribution measures to the emission reduction potential of the waste classification system. However, with the improvements in the waste classification efficiency and the recycling rate, its emission reduction advantages are no longer obvious. It accounts for less than 0.3 % of the overall emission reduction by 2060.

In the future, with the advancement of classification, the technical development of incineration and landfill will have a limited role in promoting the emission reduction of the waste classification system. The development of waste treatment methods and technologies should be inclined towards classification and recycling.

## 5 Policy implications

Reducing GHG through waste classification is a systematic project that needs to be carried out from three aspects, which are to improve efficiency, implement treatment measures adapted to waste classification, and promote resource recovery. Therefore, with the goal of carbon neutrality, several recommendations are put forward to improve the household waste classification system.

### 5.1 Ensuring the improvement of waste classification efficiency

Waste classification facilitates emission reduction in waste management systems, and enhancing the efficiency of waste classification can further amplify the benefits of the reduction. To attain carbon neutrality, it is imperative to reinforce the efficacy of waste classification. Beijing initiated waste classification pilot demonstrations in 2000 but faces challenges such as inadequate resident participation, imperfect facilities, limited recycling capacity, and policy issues [59,60], resulting in low classification accuracy with a rate of only 16.92 %–34.56 % [61]. This leaves a significant gap for improvement.

Policy support is a crucial for waste classification, as government policies exert a direct or indirect influence on waste management [62]. The primary challenge confronting our country's waste classification efforts is how to leverage policy support to enhance the efficiency of waste classification. Mandatory policies can expedite the attainment of waste classification efficiency standards. Shanghai's compulsory household waste classification policy has resulted in the effective classification of over 80 % of household waste [63]. As one of the four municipalities directly under the central government, Beijing shares commonalities and similarities with Shanghai. The successful experience of waste classification in Shanghai can be integrated with “the Regulations” to effectively promote waste classification.

### 5.2 Optimizing facilities and strategies for treatment

**Fig. 9:**
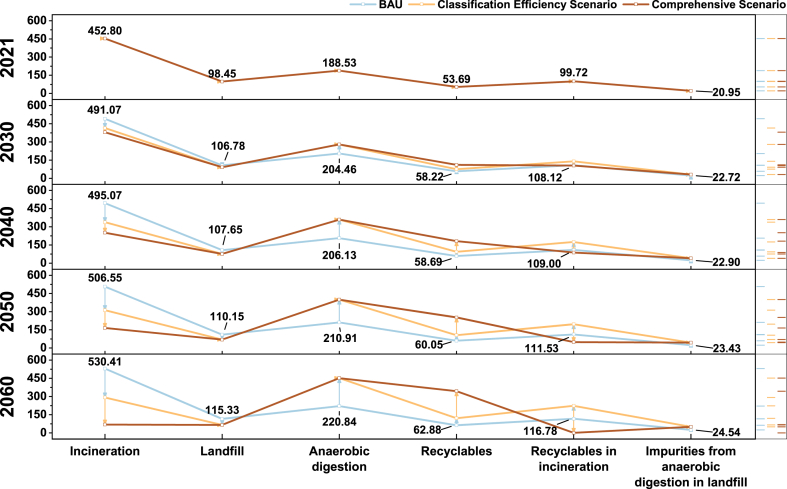
Waste volume sent to treatment facilities under three scenarios from 2021 to 2060 ( × 10^4^ t/yr). Note: 1. Incineration includes the non-recyclables portion. In the BAU scenario, 65 % of the recyclables cannot be recycled from 2021 to 2060. In the other two scenarios, theses proportions are 65.00 %, 48.75 %, 32.5 %, 16.25 %, and 0 %. This quantity of non-recyclables sent to incineration will be separately indicated in the fifth column of the figure.

Improving waste classification efficiency reduces landfill and incineration waste while increasing waste for anaerobic digestion and recycling (see Fig. 9). This promotes waste reduction, resource utilization, and recycling, impacting the emission reduction potential from treatment. Incineration power generation is widely employed due to its advantages of harmlessness, reduction, and resource utilization. Additionally, the classification of food waste can decrease waste moisture content, facilitating energy recovery in incineration [64,65]. However, as waste classification efficiency improves, the average lower calorific value (LHV) decreases, and the economic benefits of incineration diminish, limiting its applicability. Research indicates that when Beijing's waste classification rate increases from 45 % to 91 % (the separation rate of food waste and recyclables), the moisture content of incinerated waste drops from 44.42 % to 2.98 %, while its average LHV drops from 13965.12 kJ/kg to 875.85 kJ/kg (see Table 16). The average LHV of waste approaches or even falls below the minimum standard for incineration [66], requiring additional energy input. Higher waste classification efficiency compromised the operation stability of incineration, leading to reduced power generation benefits. Excessively high waste classification efficiency may not necessarily contribute to net emissions reduction [5,67]. Therefore, future efforts should focus on enhancing the technical capabilities of low calorific value waste incineration [68], while gradually shifting towards anaerobic digestion and recycling in waste management system. Additionally, greater attention should be given to anaerobic digestion and recycling, and to utilize waste more effectively rather than raw materials for new products to achieve a greater emission reduction potential and carbon neutrality goals.

2. Landfill includes impurities derived from anaerobic digestion, which accounts for 10 % from 2021 to 2060 in all three scenarios. This part will be presented separately in the sixth column of the figure.
3. The waste amount sent to anaerobic digestion is the remaining 90 % from 2021 to 2060 under all three scenarios.
4. The recyclable portion is 35 % from 2021 to 2060 in BAU scenarios. In the other two scenarios, these proportions are 35.00 %, 51.25 %, 67.50 %, 83.75 %, and 100.00 %.
5. Vertical lines with arrows indicate changes in the amount of waste treated by the four disposal methods under the three scenarios. Arrows pointing down indicate a decrease, pointing up indicate an increase, and arrows pointing right indicate no change.
6. The data label in the figure represents the data of the BAU Scenario.

**Table 16:** Moisture content (%) and average LHV (kJ/kg) of residual waste in Classification Efficiency Scenario.

	2021	2030	2040	2050	2060
Moisture content (%)	44.42	40.82	32.23	24.00	2.98
Average LHV (kJ/kg)	13965.12	12992.29	10665.96	7912.49	875.85

Data source: By calculation.

### 5.3 Establishing a waste recycling and utilization system

**Fig. 10:**
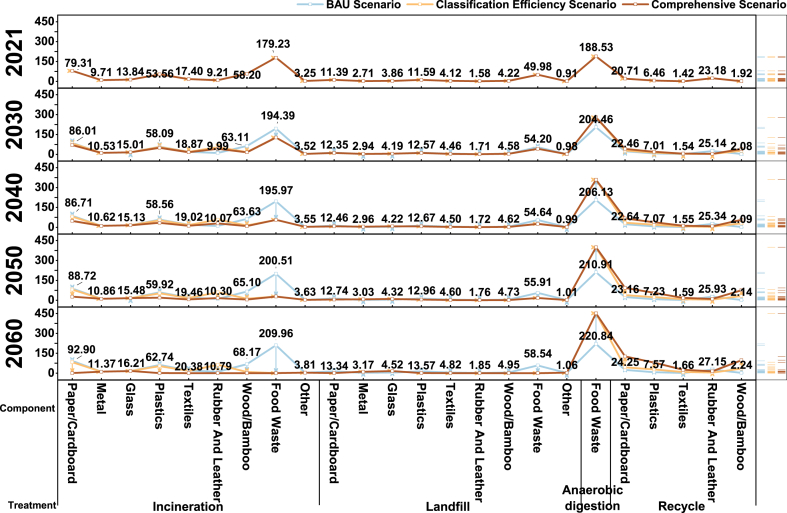
Waste volume of different components sent to treatment facilities under three scenarios from 2021 to 2060 ( × 10^4^ t/yr). Note: The data label in the k-ure represents the data of the BAU Scenario.

As waste classification efficiency improves, there are corresponding changes in the waste composition (see Fig. 10). The most significant change is observed in food waste, which constitutes a major portion of household waste in China. Additionally, four categories of recyclables, including paper/cardboard, plastics, textiles, and wood/bamboo, will also increase. Improving the recycling rate effectively increases GHG offsets for material recycling. Studies have shown that recovery rates of different components have impacted GHG emissions differently [2,20,69]. Currently, the contribution of GHG offsets can be listed in the following order plastic > wood/bamboo > paper/cardboard > food waste > textile > rubber and leather. Textiles and rubber have greater emission reduction potential through material recycling than incineration and landfill (see Table 17).

Data source: By calculation.

**Table 17:** GHG emissions offsets of six components.

Year	Components	Recycling	Incineration	Anaerobic digestion	Landfill	Total
2021	Paper/Cardboard	34.88	10.98	–	–	45.86
	Plastics	81.22	11.76	–	–	92.98
	Textiles	5.20	9.52	–	–	14.72
	Wood/Bamboo	16.18	42.66	–	–	58.84
	Rubber and Leather	7.74	6.09	–	–	13.83
	Food waste	30.55	–	7.81	–	38.36
2060-BAU Scenario	Paper/Cardboard	12.85	40.86	–	–	53.71
	Plastics	13.78	95.14	–	–	108.92
	Textiles	11.15	6.09	–	–	17.25
	Wood/Bamboo	49.96	18.95	–	–	68.91
	Rubber and Leather	7.14	9.07	–	–	16.20
	Food waste		35.79	9.15	–	44.93
2060- Classification Efficiency Scenario	Paper/Cardboard	23.38	36.47	–	–	59.86
	Plastics	51.09	80.57	–	–	131.66
	Textiles	60.12	5.08	–	–	65.20
	Wood/Bamboo	9.22	2.69	–	–	11.91
	Rubber and Leather	108.78	53.51	–	–	162.29
	Food waste	–	0.11	18.71	–	18.82
2060- Comprehensive Scenario	Paper/Cardboard	66.81	0.79	–	0.11	67.71
	Plastics	145.96	2.78	–		148.74
	Textiles	171.78	0.19	–	0.02	172.00
	Wood/Bamboo	26.34	0.19	–	0.05	26.57
	Rubber and Leather	310.80	0.21	–	0.02	311.03
	Food waste	–	0.19	19.41	0.03	19.63

With the increasing proportion of recyclable resources in household waste, it has considerable utilization value for resource utilization [60]. This study confirms the GHG reduction benefits of promoting recycling and resource utilization through waste classification. However, the implementation of waste classification may result in the loss of economic benefits for incineration enterprises, and recycling lacks cost competitiveness compared with raw materials usage [70]. Therefore, the reuse of recyclables based on waste classification is strongly opposed by stakeholders [59]. Chen (2016) proposed that “polluter pays” has promoted Taiwan's recycling industry development. Beijing can learn from Taiwan's experience and develop a suitable plan for the city to ensure sustainable waste classification. Additionally, encouraging and mandatory policies have different impacts on the effectiveness of waste classification [71]. Encouraging policies such as cognition training, normative constraints, publicity and education, and improved facilities and service can directly influence citizens' behavioral decisions and willingness to participate in waste classification [72]. Economic measures can boost waste classification efficiency. For recyclables with high economic value and strong emission reduction potential, measures such as social funds and market-oriented management can promote their utilization. For those with low economic value but who have a strong contribution to emission reduction, non-market methods such as financial subsidies and carbon tax support can be provided to ensure its reuse.

## 6 Conclusions

This study compares the GHG emissions between the traditional mixed collection and transportation system and the waste classification system, and explores the net emissions of the waste classification system in Beijing from 2021 to 2060, considering reduction potential from the improved classification system and advanced technology. The results indicate that waste classification can lower the net emissions of the entire waste system, and its GHG reduction potential depends on the waste flow, recycling, and treatment strategies. The order of net emission intensity of the four methods is landfill > incineration > anaerobic digestion > recycling. The future development of waste treatment should be inclined to recycling.

To further study the emission reduction potential of Beijing's waste classification system, three scenarios are set up and the GHG offset of seven measures are compared. The Classification Efficiency Scenario shows a significant and sustained reduction in net emissions compared to the BAU Scenario. Among the seven measures, improving waste classification efficiency and recycling rate are the most effective measures to reduce net emissions.

Waste classification has significant benefits for reducing GHG emissions, and source classification also enables the emission reduction potential from recycling. It should be noted that increasing recycling only reduces the embodied carbon emissions of raw materials rather than reducing net emissions from their production. Changes in waste flow, composition, moisture content, and calorific value, coupled with improved waste classification efficiency, will affect the existing waste management strategies. GHG emissions from incineration will inevitably increase, and waste management strategies that prioritize recycling are crucial for realizing potential climate benefits. Furthermore, the emission reduction potential of various components in the recycling process varies. Attention should be paid to the recyclables with high economic value and strong emission reduction potential, and the recyclables with low economic value but a great contribution to emission reduction should be guaranteed through government support. Policymakers should take into account the emission reduction potential from the classification system. Through the joint efforts of the government, enterprises, and the public, waste classification can be further consolidated to achieve emission reduction targets.

## Data availability statement

The data used in the article comes from publicly available sources, which had been listed in the manuscript and supplementary materials. Data will be made available upon request.

## CRediT authorship contribution statement

**Zhixin Wen:** Writing – original draft, Visualization, Validation, Software, Methodology, Formal analysis, Data curation. **Huimin Li:** Writing – review & editing, Resources, Project administration, Funding acquisition, Conceptualization. **Yufei Wang:** Writing – review & editing, Validation, Resources. **Xiaofan Zhao:** Supervision, Project administration, Investigation. **Xianghui Deng:** Supervision, Resources, Investigation.

## Declaration of competing interest

The authors declare that they have no known competing financial interests or personal relationships that could have appeared to influence the work reported in this paper.
